# Gynecological cancers and the global COVID-19 pandemic

**DOI:** 10.4274/jtgga.galenos.2020.2020.0119

**Published:** 2020-12-04

**Authors:** Ibrahim Alkatout, Mojgan Karimi-Zarchi, Leila Allahqoli

**Affiliations:** 1 Department of Obstetrics and Gynecology, University Hospitals Schleswig-Holstein, Kiel School of Gynaecological Endoscopy, Kiel, Germany; 2 Department of Gynecology Oncology, Firoozgar Hospital, Iran University of Medical Sciences, Tehran, Iran; 3 Iran University of Medical Sciences, Tehran, Iran

**Keywords:** COVID-19, gynecological cancer, oncology, elective surgery, chemotherapy, radiotherapy

## Abstract

Coronavirus disease-2019 (COVID-19) has reduced the availability of health resources which will affect treatment of gynecological cancers. The present study aimed to provide a treatment protocol for patients with gynecological cancers during the global COVID-19 pandemic. International databases with keywords of COVID-19; Severe Acute Respiratory Syndrome; Middle East Respiratory Syndrome; gynecologic cancer; cervical cancer; and vaginal cancer, vulvar cancer, ovarian cancer, endometrial cancer, tumor, elective surgery, chemotherapy, radiotherapy, cancer, guideline, guidance, women, management, outpatient clinic visits, and triage were comprehensively searched. All the obtained guidelines were studied and the contents were summarized. During the COVID-19 pandemic, early stage endometrial cancer was preferably treated with hormone therapy while radiotherapy was given in preference in later stages. Cervical intraepithelial neoplasia 3 and high-grade squamous intraepithelial lesions should be treated immediately after diagnosis using at least a loop electrosurgical excision procedure while any major surgery should be postponed by 10-12 weeks. In the early stage of cervical cancer, surgery may be delayed by 2-4 weeks, and radiotherapy prescribed for the intervening period. In cases of an ovarian mass with negative tumor markers, no sign of cancer on imaging investigations, no ascites, a low serum CA-125 level, and no papillary projection or vegetation in the base of the cyst, the patient may be given hormone therapy for 2-3 months. In cases of newly diagnosed confirmed ovarian cancers, surgery should be performed as early as possible (maximum: 2-3 weeks). Vulvar and vaginal cancers can be treated within 10-12 weeks of diagnosis, but radiotherapy should be given in preference in this situation. A molar pregnancy is an oncological emergency for which a suction curettage is mandatory; the patient must be monitored for metastases. Information concerning the choice between open or laparoscopic surgery is limited. Given that any patient may be an asymptomatic carrier of the coronavirus, major surgery should be preceded by chest computerized tomography, with and without contrast medium, in order to detect lung lesions. Evidence concerning these recommendations is limited because of the novel and unknown nature of the COVID-19 pandemic. Furthermore, data pertaining to ethical debates about delayed treatment and treatment approaches deviating from current guidelines are also limited.

## Introduction

The coronavirus is a major pathogen that appears to primarily targets the human respiratory system. Previous outbreaks of coronaviruses (CoVs) include the Severe Acute Respiratory Syndrome-CoV (SARS-CoV) and the Middle East Respiratory Syndrome-CoV (MERS-CoV), which were identified as a major threat to public health (1). Older adults and persons of any age with a serious underlying medical condition are at high risk of severe illness from Coronavirus disease-2019 (COVID-19) (2). Treatment for gynecological cancer may weaken the immune system (immunocompromised) and this makes the patients a “high-risk” group for severe effects of COVID-19 (3,4). We lack sufficient information about the diagnosis of cancer, its surgical treatment, chemotherapy, and radiotherapy in patients with an immune deficiency during the global COVID-19 pandemic (5). In addition, medical staff are not trained in counseling patients about the subject. Ethical issues concerning delayed treatment and using therapeutic approaches that deviate from current guidelines are also unresolved (6). The benefits of delaying or modifying treatment must be weighed against the risks of proceeding with regular treatment (3,7,8,9,10). Future studies in cancer patients during any emergency situation that availability of health care resources is limited are still vital (5,9). The aim of the current review was to address all existing protocols concerning this issue and provide a well-rounded approach towards the treatment of patients with gynecological cancers during the COVID-19 global pandemic or any similar emergency situation.

## Data sources

We performed a comprehensive review of international databases, including Science Direct, PubMed, Cochrane Library, Scopus, and Google Scholar. Keywords used in the searches were: COVID-19; SARS; MERS; gynecologic cancer; cervical cancer; and vaginal cancer, vulvar cancer, ovarian cancer, endometrial cancer, tumor, elective surgery, chemotherapy, radiotherapy, cancer, guideline, guidance, women, management, outpatient clinic visits, and triage. In view of the limited period of time since the start of the COVID-19 pandemic and the absence of extensive information, we took the early guidelines published in various countries and expert opinions into account. The current recommendations are based on existing evidence and may need to be updated as more information or national/international guidelines become available.

## General planning of cancer treatment during the COVID-19 pandemic

- The following criteria apply to cancer patients with a high risk of severe complications during the pandemic: age ≥65 years, patients of any age with cardiovascular or pulmonary disease or diabetes, an Eastern Cooperative Oncology Group status ≥2, and those receiving systemic chemotherapy (11).

- In general, it would be reasonable to perform surgery when the patient’s survival is expected to be >12 months, the patient does not respond to other alternative treatments, and survival would be compromised if surgery were to be delayed (12,13).

- Whenever non-surgical approaches such as radiotherapy or neoadjuvant chemotherapy can be used instead of surgery, it would be advisable to delay surgery, provided the patient has ready access to the intensive care unit (ICU) and other hospital facilities (14).

- Elective operations for benign conditions should not be performed during the pandemic. If possible, the patients should be given alternative medical treatment to minimize their symptoms and encouraged to stay at home rather than in the hospital (15).

- In times of crisis, healthcare providers should be able to focus their attention and resources on the care of persons severely affected by the coronavirus (15).

- Legally, the patients and their families must be fully informed about the delay in surgery or the use of non-surgical treatment, and the circumstances must be carefully recorded in the informed consent form (12,13).

- Decisions should be made on the basis of weekly tumor boards by a multidisciplinary team (MDT) (12,13,16).

- Both medical staff and patients are at risk of being infected by COVID-19 during cancer treatment (15).

## Outpatient management and gynecological oncology clinics

- Patients should be screened on the telephone about COVID-19 symptoms. At the subsequent personal meeting, their body temperature should be measured if possible (12).

- Visits should be restricted to new patients, absolutely essential consultations to address acute oncologic issues, and patients undergoing active treatment for their disease (molar pregnancies and symptomatic patients with cancer recurrence) (12,17,18).

- In cases of patients residing in a different town or city, imaging and laboratory studies should be performed at their residential locations and sent to the treating physician electronically, who then decides about the appropriate treatment (12).

- The number of persons accompanying the patient should be reduced to one. Furthermore, when the patient needs help due to physical or psychological limitations, it should be ensured that the accompanying person is not suspected of having COVID-19 and is not in contact with any person suspected of being infected by the virus (12,17).

- Physical distance should be maintained in the waiting room. The attendance of patients should be planned carefully to prevent crowding (12,13).

- All routine follow-up/surveillance visits should be postponed, or the consultations should be performed via telemedicine or the Internet - if resources permit - until the crisis has stabilized and one may return to the usual operating procedures (18).

- Screening procedures such as mammography and Pap smears should be delayed. If the patient needs to be followed up (within a period of 3 to 6 months), one should opt for the outer time limit (6 months) (12).

- Any intervention that is not absolutely essential should be postponed, such as routine imaging studies or serum markers, in patients who are asymptomatic and have no evidence of disease at the most recent evaluation (17).

## Management of gynecological oncologic diseases

The National Health Service in England states that individual patient decisions must be made by MDTs (14,15). Patients should be prioritized for surgery on the basis of age, comorbidities, family history of cancer, physical aspects, radiological findings, tumor markers (19), and the risk of needing ICU treatment (17). The priority of timing of gynecological cancer surgery under pandemic conditions (COVID-19) or any emergency situation is summarized in Table 1.

## Specific considerations for the treatment of gynecological cancer during COVID-19

Surgery should be postponed during the COVID-19 pandemic because of the risks of surgery, anesthesia, the possibility of the patient being an asymptomatic carrier, and developing symptoms postoperatively. It will be difficult to determine whether the complications and lung lesions are caused by the coronavirus or by surgery (4). The existing evidence indicates that, during the COVID-19 pandemic and other global emergencies, clinicians will have to align their treatment of gynecological cancers to the risk-benefit ratio. Specific considerations for treatment during the COVID-19 pandemic or any emergency situation are summarized in Table 2.

## General recommendations for surgery during the COVID-19 pandemic:

- Make sure you speak to the patient about the possibility of a COVID-19 infection and its consequences (12).

- The virus could be transmitted to the staff during open surgery or laparoscopy (12).

- The patient should undergo a COVID-19 test before surgery (7,15). In the event of a positive test, surgery should be postponed to after recovery (17).

- During the COVID-19 pandemic, any oncology surgery with the risk of bleeding, infection, and the possibility of requiring ICU should be postponed until appropriate conditions prevail and the required facilities are available (20).

- If possible, intubation and extubation should be performed in a room with negative pressure (12).

- Operating rooms used for patients with suspected COVID-19 should be separate from those for other patients and should be properly ventilated. If possible, patients with COVID should be given operating rooms with negative ventilation (15).

- Only the main staff should be involved in the surgery, except in emergencies. Staff should not be replaced during the operation (13).

- All employees should use personal protective equipment (PPE), such as gowns and protective shields. Wearing and removing PPE must be performed fully in accordance with the existing health recommendations (12).

- Electrosurgery tools should be used with the minimum power setting of the device, and their use should be minimized because they generate particle aerosols.

- Surgical instruments used in patients with suspected COVID-19 should be washed and sterilized separately from other instruments.

- In persons with suspected or confirmed COVID-19, procedures that cause aerosols, such as intubation and extubation, bag-masking, or electrocautery, should be performed under full personal protection (PPE, including mask N95).

- Operating theatres used for emergency surgery should be separated from that of elective surgeries (12).

- Information concerning the preferred choice between open or laparoscopic surgery is limited. In gynecological emergencies and cancer, laparoscopic surgery would be advantageous to the health system in terms of reducing the duration of hospital stays (12). The release of CO_2_ should be minimized during laparoscopic surgery (15). The approach that provides maximum safety to the patient and staff and also ensures the shortest operating time should be given preference.

- Given that patients with gynecological cancer may be asymptomatic carriers of the coronavirus, it is best if major surgeries, such as ovarian and endometrial cancer, a real-time reverse transcription polymerase chain reaction (rRT-PCR) test from a throat swab or preferably computed tomography (CT)-thorax with and without contrast should be performed to diagnose lung lesions (20).

- Evidence about COVID-19 is still limited because of the novel and unknown nature of the disease. Medical staff (doctors, nurses, technicians, etc.) as well as patients lack clear data on the subject (21).

## Conclusion

- Any delay in gynecological procedures that could exert a negative effect on the patient’s health and safety should be avoided. Obstetricians, gynecologists, and other health care practitioners should be aware of the unintentional impact of policies regarding COVID-19, including limited access to time-sensitive obstetric and gynecological procedures.

- Cancer patients bear a higher risk from infection with SARS-CoV-2 than the general population. The risk of severe respiratory complications is high in cancer patients with SARS-CoV-2, who may then require treatment in the ICU.

- The risk of respiratory complications is associated with a history of chemotherapy or surgery in the month preceding the COVID infection (this factor concerns the large majority of cancer patients).

- In cases of life-threatening diseases such as severe hemorrhage in endometrial cancer, surgery - if possible by the minimally invasive approach - should be performed as early as possible.

- In cases of molar pregnancies or newly recognized ovarian cancers, which are gynecological emergencies, surgery is best performed by experienced oncologists; the surgeon should select the simplest type of operation with the minimum operating time and complications.

- In non-emergency cancers, surgery may be delayed for at least 10-12 weeks.

- In cases of cancers that can be treated with radiotherapy, the latter should be started and surgery postponed.

- Given that patients with gynecological cancer may be asymptomatic carriers of the coronavirus, any major surgery such as that for ovarian or endometrial cancer should be preceded by a rRT-PCR test, or preferably a CT of the chest with and without contrast in order to detect lung lesions.

- Ethical issues concerning the use of treatment that differs from current guidelines warrant further investigation.

## Figures and Tables

**Table 1:**
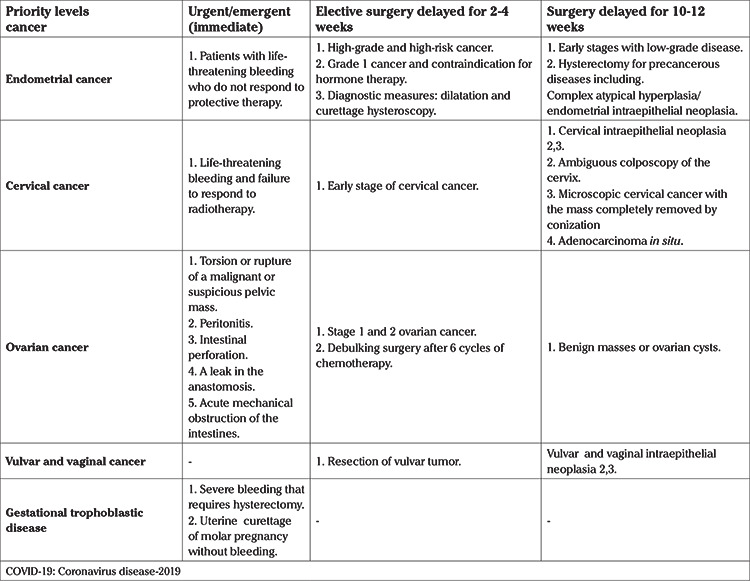
The priority of surgical timing in patients with gynecological cancer during the COVID-19 pandemic (12)

**Table 2:**
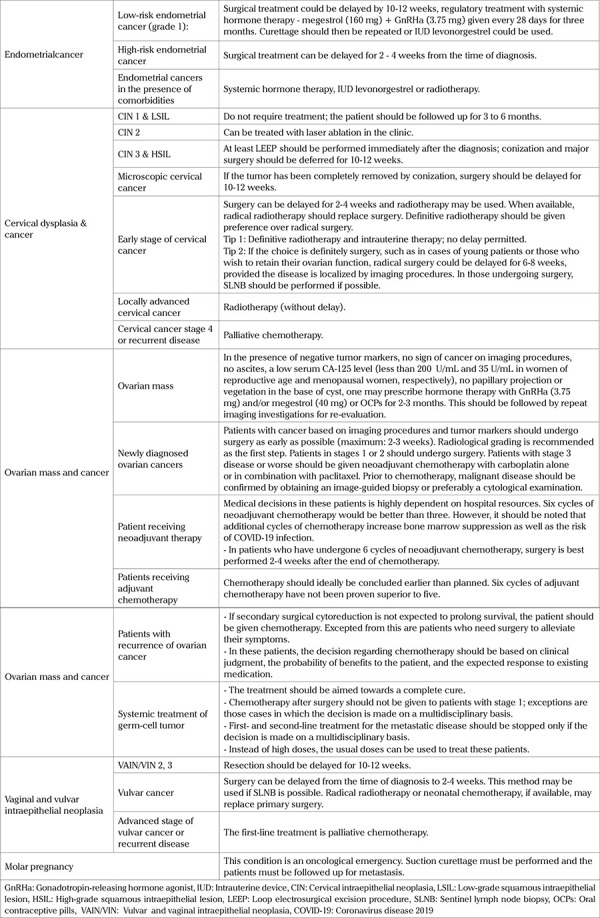
Specific considerations for the treatment of gynecological cancer during the COVID-19 pandemic (7,12,13,14,15,16,17,20,22,23,24,25,26,27,28,29)
